# Dengue Virus Type 2 in Travelers Returning to Japan from Sri Lanka, 2017

**DOI:** 10.3201/eid2311.171293

**Published:** 2017-11

**Authors:** Motoyuki Tsuboi, Satoshi Kutsuna, Takahiro Maeki, Satoshi Taniguchi, Shigeru Tajima, Fumihiro Kato, Chang-Kweng Lim, Masayuki Saijo, Saho Takaya, Yuichi Katanami, Yasuyuki Kato, Norio Ohmagari

**Affiliations:** National Center for Global Health and Medicine, Tokyo, Japan (M. Tsuboi, S. Kutsuna, S. Takaya, Y. Katanami, Y. Kato, N. Ohmagari);; National Institute of Infectious Diseases, Tokyo (T. Maeki, S. Taniguchi, S. Tajima, F. Kato, C.-K. Lim, M. Saijo)

**Keywords:** dengue fever, Sri Lanka, outbreak, travelers, viruses, dengue virus, Japan, vector-borne infections, mosquitoes

## Abstract

In June 2017, dengue virus type 2 infection was diagnosed in 2 travelers returned to Japan from Sri Lanka, where the country’s largest dengue fever outbreak is ongoing. Travelers, especially those previously affected by dengue fever, should take measures to avoid mosquito bites.

In 2009, Sri Lanka experienced an outbreak of dengue fever, which was the largest since dengue fever was classified as a reportable disease in 1996. During that outbreak, 35,008 dengue fever cases and 346 related deaths were reported (*1*). The outbreak and severe dengue were attributed to the new dengue virus type 1 (DENV-1) strain (*1*), which has since remained the predominant serotype in Sri Lanka (*2*).

However, the number of patients with dengue fever has increased drastically during the current and largest outbreak, during January–July 2017, when >90,000 patients were reported in Sri Lanka, particularly in Colombo (*3*). In this outbreak, little is known about the causative virus type. We describe 2 travelers from Japan who were infected with DENV-2 during a late June 2017 visit to Sri Lanka.

In late June 2017, a previously healthy 34-year-old Japanese man (case-patient 1) sought care at the National Center for Global Health and Medicine (Tokyo, Japan) with a 2-day history of a high-grade fever, headache, fatigue, mild stomach ache, and watery diarrhea. His symptoms had begun the day after he returned to Japan after ≈2 months in Colombo, Sri Lanka. He had been bitten by mosquitoes in Colombo. Upon examination, his temperature was 38.9°C, and he had no abnormal findings except congested bulbar conjunctiva. Erythema appeared on his trunk and extremities on day 4 after fever onset. Nonstructural protein antigen positivity (negative for DENV IgM and IgG) and detection of the DENV-2 genome in his serum sample by real-time reverse transcription PCR (rRT-PCR) (cycle threshold 27.8) confirmed dengue fever.

In late June 2017, a previously healthy 56-year-old Japanese woman (case-patient 2) visited the National Center for Global Health and Medicine with a 4-day history of high-grade fever, headache, and arthritis and 3-day history of watery diarrhea. She had visited Colombo for 5 days, and her symptoms began 3 days after she returned to Japan. She also had been bitten by mosquitoes. Upon examination, her temperature was 37.2°C, and she had slight erythema on her face and trunk. Dengue fever was diagnosed on the basis of nonstructural protein antigen positivity (negative for DENV IgM and IgG) and detection of the DENV-2 genome in her serum by rRT-PCR (cycle threshold 23.2).

We amplified virus genome obtained from patients’ serum by rRT-PCR and sequenced the E protein coding region of the DENV-2 genome, which revealed that both strains (GenBank accession nos. LC312196 [case-patient 1] and LC312197 [case-patient 2]) belonged to the Cosmopolitan genotype of DENV-2 and shared 99% identity with DENV-2 strains isolated in Singapore in 2014 (accession nos. KX224269 and KX224268) and China in 2015 (accession no. KU504492) (Figure). A phylogenetic tree based on the envelope region of the DENV-2 genome revealed that these 2 isolates belonged not to the branch of Africa strains but to the lower branch, which comprised Asia isolates within the Cosmopolitan genotype.

**Figure:**
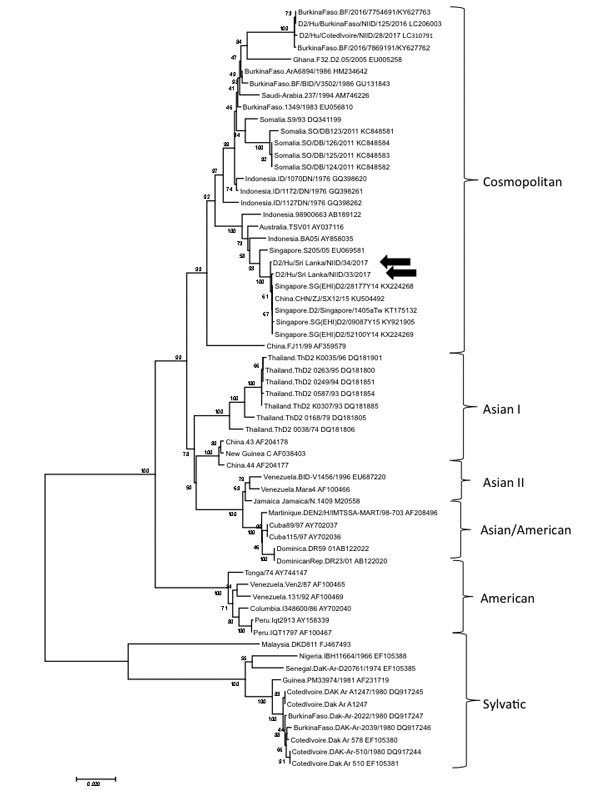
Phylogenetic analysis of dengue virus type 2 strains obtained from 2 patients who returned to Japan from Sri Lanka in June 2017 (arrows) and a comparison with reference sequences from GenBank. Virus lineages are shown at right. Phylogenetic tree was constructed by using the neighbor-joining method. The maximum composite likelihood method was used, and the rates among sites were uniform. These analyses were performed using MEGA7 (http://www.megasoftware.net). Scale bar indicates nucleotide substitutions per site.

Dengue fever was first serologically confirmed in Sri Lanka in 1962 (*4*). Since then, although all 4 serotypes (DENV-1–4) were present, epidemics caused by DENV-3 in 1989 and 2002–2004 and by DENV-1 in 2009 were reported nationwide (*1*). After the outbreak in 2009, the annual number of dengue fever patients remained stable at 30,000–50,000 (*3*) (Technical Appendix Figure). However, the present outbreak situation is more serious because the number of patients has already exceeded the average number of cases during the same interval by >3.5-fold and continues to increase (Technical Appendix Figure). The Ministry of Health of Sri Lanka reported 93,322 dengue fever cases, including 250 deaths, as of July 17, 2017 (*5*). Our findings indicate that case-patients 1 and 2 were infected with DENV-2 Cosmopolitan genotype during the same period, suggesting that it might be the causative strain in the worst-ever outbreak of this disease in Sri Lanka. 

The epidemic strain was highly related to the strains from Southeast Asia because the sequences in current cases were nearly identical to that of a DENV strain isolated in Singapore in 2014 and in Zhejiang Province, China, in 2015. As previously reported, DENV-2 and DENV-3 are associated with severe disease accompanying secondary dengue infections (*6*,*7*), suggesting that the current epidemic of dengue fever could be the worst fatal outbreak in Sri Lanka.

In summary, we report 2 travelers from Japan infected with DENV-2 in Sri Lanka, where the largest reported outbreak in the country’s history began in January 2017. Because the virulent DENV-2 strain is considered the causative agent in this epidemic and the number of deaths has been increasing, we encourage travelers, particularly those who have been previously affected by dengue fever, to prepare against vector mosquitos (e.g., by properly using insect repellents) to avoid DENV infection.

Technical Appendix Distribution of reported dengue fever cases, Sri Lanka, 2010–2017
