# Supplementary material for: Dengue Virus Type 2 in Travelers Returning to Japan from Sri Lanka, 2017
Source: Emerg Infect Dis. 2017 Nov;23(11):1931–3. doi: 10.3201/eid2311.171293 (PMC5652411; doi:10.3201/eid2311.171293)
Supplement: Technical Appendix — Distribution of reported dengue fever cases, Sri Lanka, 2010–2017 [file 17-1293-Techapp-s1.pdf]

Article DOI: https://doi.org/10.3201/eid2311.171293

Dengue Virus Type 2 in Travelers Returning to Japan from Sri Lanka, 2017

Technical Appendix

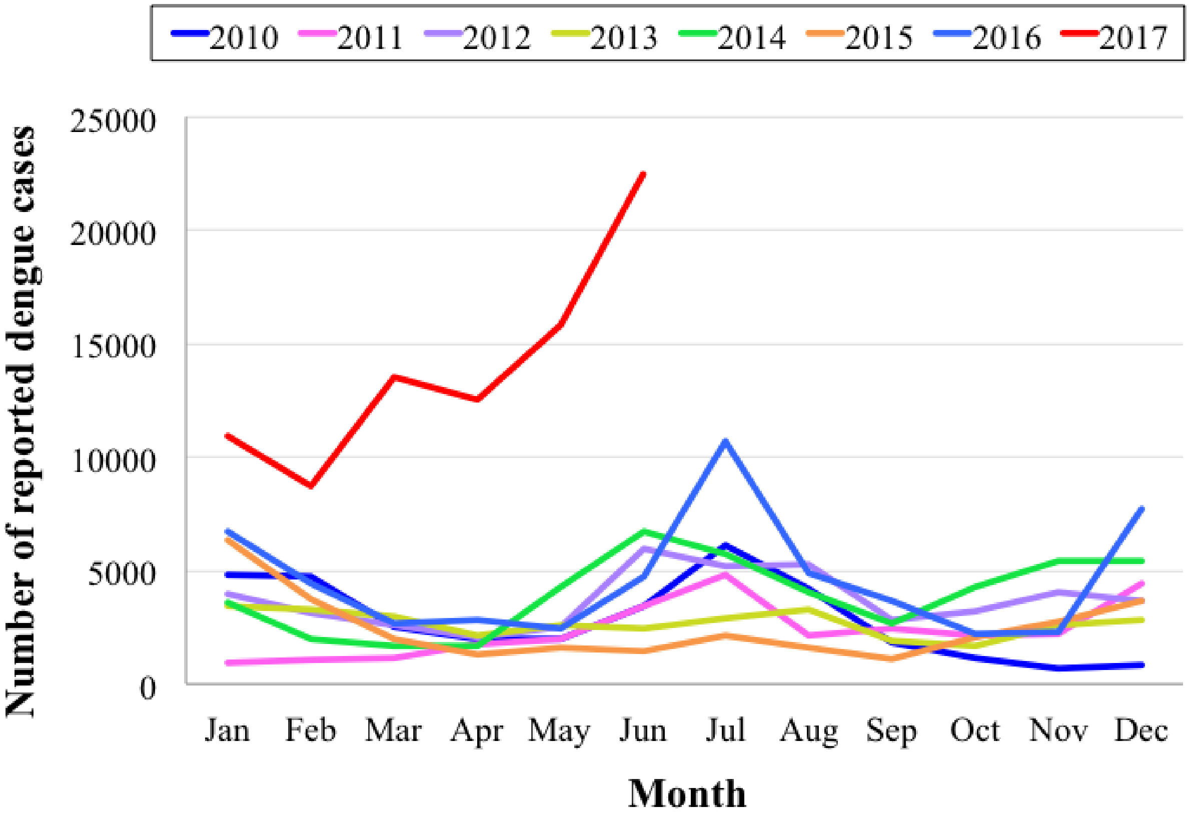

Technical Appendix Figure. Distribution of reported dengue fever cases, Sri Lanka, 2010–2017. Figure generated using data from the Epidemiology Unit, Ministry of Health, Sri Lanka (http://www.epid.gov.lk/web/index.php?option=com_casesanddeaths&Itemid=448&lang=en).
